# Characterization of immune cell migration using microfabrication

**DOI:** 10.1007/s12551-021-00787-9

**Published:** 2021-02-11

**Authors:** Doriane Vesperini, Galia Montalvo, Bin Qu, Franziska Lautenschläger

**Affiliations:** 1 Department of Experimental Physics, Saarland University, 66123 Saarbrücken, Germany; 2 Center for Biophysics, Saarland University, 66123 Saarbrücken, Germany; 3 Biophysics, Center for Integrative Physiology and Molecular Medicine (CIPMM), School of Medicine, Saarland University, 66421 Homburg, Germany; 4 Leibniz Institute for New Materials, 66123 Saarbrücken, Germany

**Keywords:** Immune cells, Amoeboid migration, Microfabrication, Target search

## Abstract

The immune system provides our defense against pathogens and aberrant cells, including tumorigenic and infected cells. Motility is one of the fundamental characteristics that enable immune cells to find invading pathogens, control tissue damage, and eliminate primary developing tumors, even in the absence of external treatments. These processes are termed “immune surveillance.” Migration disorders of immune cells are related to autoimmune diseases, chronic inflammation, and tumor evasion. It is therefore essential to characterize immune cell motility in different physiologically and pathologically relevant scenarios to understand the regulatory mechanisms of functionality of immune responses. This review is focused on immune cell migration, to define the underlying mechanisms and the corresponding investigative approaches. We highlight the challenges that immune cells encounter in vivo, and the microfabrication methods to mimic particular aspects of their microenvironment. We discuss the advantages and disadvantages of the proposed tools, and provide information on how to access them. Furthermore, we summarize the directional cues that regulate individual immune cell migration, and discuss the behavior of immune cells in a complex environment composed of multiple directional cues.

## Migration of immune cells is central for immune surveillance

**Fig. 1:**
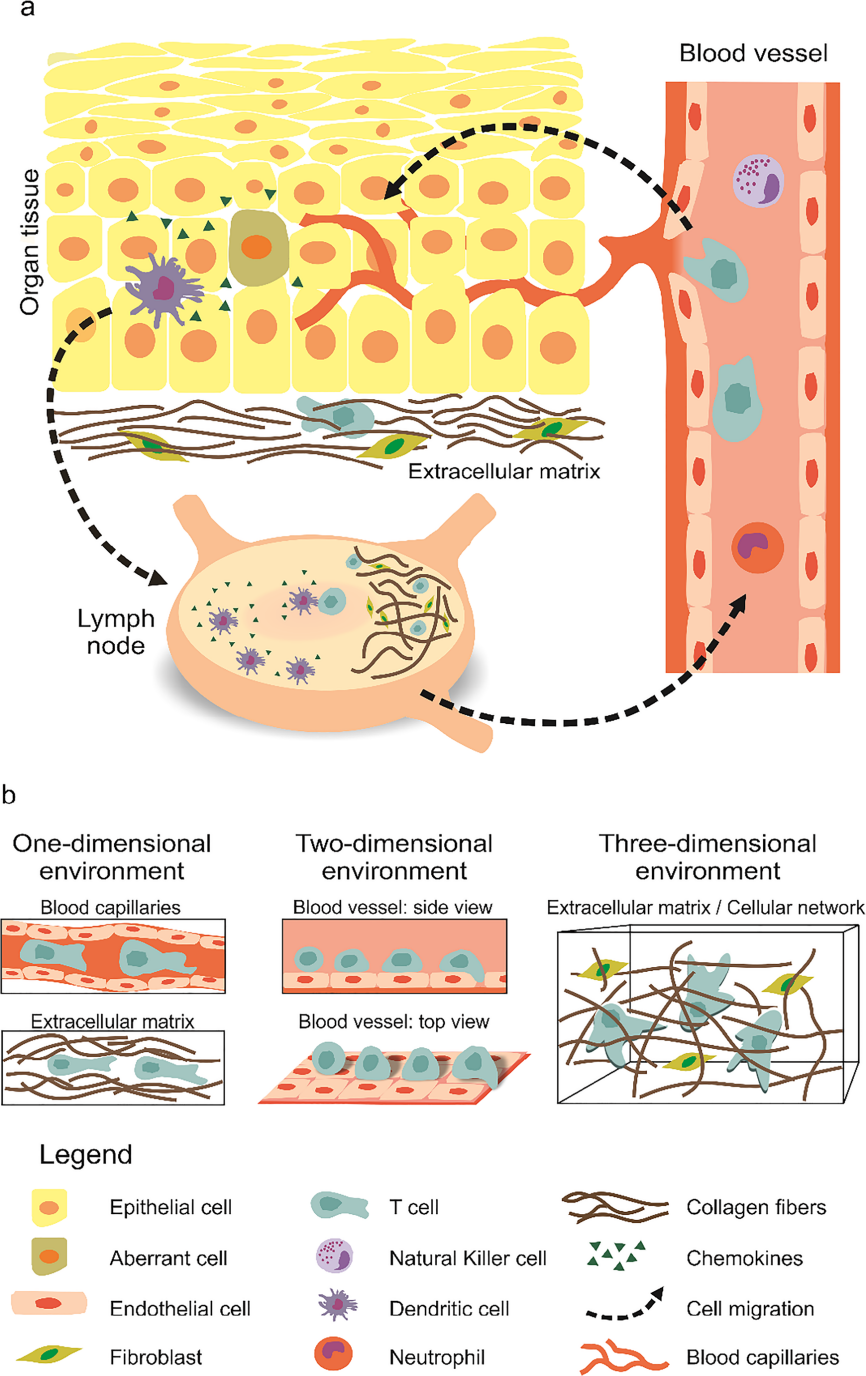
Immune cell migration in vivo and the diverse scenarios encountered. a) Overview of immune cell migration in vivo. From blood vessels, immune cells transmigrate into and then patrol peripheral tissues/organs to clear invaders and/or collect antigens. Then, immune cells enter lymph vessels and migrate toward the lymph nodes. Peripheral dendritic cells (DCs) are responsible to collect antigens from aberrant (infected or malignant) cells in peripheral tissues (skin is shown here). Upon recognition of an invader or aberrant cells, an immediate immune response is initiated locally. Then, professional antigen-presenting cells (APCs) go to the lymph nodes, where the adaptive immune cells (B and T cells) are activated. Activation is then followed by proliferation. Effector cells enter the blood circulation and transmigrate into the respective inflammation sites. b) Schematic of 1D, 2D and 3D scenarios encountered by immune cells during migration. T cells are drawn here in the illustration as an example of immune cells. 1D is found in blood/lymph capillaries and in the cavities/channels in ECM. The blood vessel walls, which immune cells are rolling on, correspond to a 2D scenario. In general, while patrolling the tissues, immune cells face a 3D environment with ECM as a main component

From the early stages in the development of the immune system, precursors of immune cells migrate from bone marrow to the thymus and to secondary lymphoid organs to continue their differentiation, or to specific tissues to become resident sentinel cells (Germain et al. 2012). When an infectious agent enters the body, two lines of defense can be activated: innate immunity and adaptive immunity. Innate immunity is a rapid immune response that is initiated within minutes after intrusion of a pathogen, without any specific pre-activation. Adaptive immunity, on the other hand, is antigen-dependent and generates immunological memory (Marshall et al. 2018). In general, immune surveillance is dependent on the constant traffic of immune cells, in terms of their migration through the blood and lymphatic systems. From there, they can be recruited to sites of tissue damage or infection, and fine-tune their effector properties in specific secondary lymphoid organs (Fig. 1a).

Innate immune cells arrive first at inflammation sites, and while killing pathogens to resolve any infection, they release cytokines (including chemokines) that recruit other innate and adaptive immune cells. Some specialized innate immune cells, such as dendritic cells (DCs), collect the antigens at inflammation sites and then migrate back to the secondary lymphoid organs to trigger activation of adaptive immune cells (de Winde et al. 2020). Neutrophils activate a rapid migratory response, which means that they are among the first innate immune cells to arrive at a site of inflammation when a pathogen enters the body. Neutrophils can also then re-enter the vasculature, in a process termed “reverse transendothelial migration” (de Oliveira et al. 2016).

Natural killer (NK) cells are essential players for the elimination of pathogen-infected or tumorigenic cells in an antigen-independent manner. Although the recirculation and movement of NK cells among human organs are not yet fully understood (Di Vito et al. 2019), it is well accepted that NK cells not only populate the peripheral blood, but also reside in almost every tissue and organ. This suggests that these NK cells can either migrate and reside in tissues, or they can constantly recirculate through the organs (Di Vito et al. 2019).

Dendritic cells are professional antigen-presenting cells that link the innate and adaptive immune systems. DCs migrate through different tissues and across many barriers; they leave the bone marrow and travel through the blood to seed all organs and tissues (de Winde et al. 2020). When a pathogen enters, the tissue-resident immature DCs at the site of inflammation collect and process the antigenic material. Similarly, DCs can detect tumor antigens and take them to the secondary lymph nodes to activate the adaptive immune responses against cancers (Nourshargh and Alon 2014). As a result of their stimulation, DCs further differentiate to a mature phenotype with up-regulated chemokine receptors. These mature DCs leave the inflammatory site and return to the draining lymph nodes to activate T cells and B cells (de Winde et al. 2020).

Together with B cells, T lymphocytes represent the adaptive immune system (Garcia 2019). The life cycle of T cells starts in the bone marrow, continues in the thymus, and then throughout the body, until they encounter their specific target cells (Krummel et al. 2016). This encounter can occur in lymph nodes, where T cells are activated by the professional antigen-presenting cells. Before activation, naïve T cells show random migration and speed fluctuations, as they alternate between periods of fast and slow movements (Krummel et al. 2016). This mode of migration allows individual T cells to examine a large area of a lymph node (Moreau and Bousso 2014). Suboptimal stimulation is considered to be the physiological trigger for T cells to change the direction of their migration more frequently and hence to keep searching. When T cells encounter matching antigen-presenting cells, they halt and establish an immunological synapse with the target cells (Moreau and Bousso 2014; Moreau et al. 2015). Upon activation, T cells change their migration program and start to move from the lymph nodes to the corresponding “battle field” in the peripheral tissues (Lämmermann and Germain 2014). To carry out their killing functions, cytotoxic T lymphocytes (mainly as activated T cells) use cytotoxic granules or the Fas/FasL pathways to destroy infected cells or tumor cells (Barry and Bleackley 2002). Promotion of migration of these cytotoxic T lymphocytes results in their higher killing efficiency (Schoppmeyer et al. 2017).

### Immune cell migration modes

The different geometries that immune cells encounter in vivo, together with their intrinsic properties, determine their migration modes. In general, cell migration can be classified into two modes: mesenchymal and amoeboid migration (Liu et al. 2015; Moreau et al. 2018). Mesenchymal migration is characterized by strong adhesion sites, proteolytic degradation of the extracellular matrix (ECM), elongated cell shape with long membrane protrusions, and slow cell movement. This type of movement mostly describes the behavior of epithelial-derived and cancer cells, rather than immune cells. For amoeboid migration, although this classification is still under constant review and varies across studies, several common aspects are widely accepted: low cell adhesion, independence from proteolytic degradation of the ECM, and rounded cell morphology with a highly contractile rear part, known as the uropod (Renkawitz et al. 2009). In vivo, immune cells mostly use the amoeboid mode of motility. The migration speed of immune cells is not constant, but varies between fast (~20 μm/min) and slow (<1 μm/min) migration phases (Chabaud et al. 2015). Such a migration pattern has been described in theoretical studies of intermittent search behavior, and it thus helps immune cells to optimize their broad space exploration and direct their migration to inflammatory sites (Bénichou et al. 2006; Bénichou et al. 2011; Petrie et al. 2009). Interestingly, the different cell types are not absolutely committed to either mesenchymal or amoeboid migration, as they can transition between these states. The mechanism behind this transition appears to be dependent on the activation status of the cells, their physiological context, their interactions with the ECM, and their adaptation to the cellular environment (Huse 2017; Liu et al. 2015). A generic model to explain migration transitions indicates the relevance of these two parameters: the intrinsic properties of the cells, and the environmental characteristics (Liu et al. 2015).

### Regulation of immune cell migration

Cell migration can be broken down into various steps, which include polarization, protrusion in the direction of motion, adhesion, translocation of the cell body, and retraction of the uropod (Mayor and Etienne-Manneville 2016). The proportion and relevance of each step depends on the migration mode, the experimental conditions, and the cell type. More specifically, polarization as the first step refers to the formation of a stable front and rear for migrating cells. For immune cells, the polarity might be an intrinsic property, like the ability of neutrophils to self-polarize (de Oliveira et al. 2016). However, polarization can also be induced by stimuli, such as chemotactic or mechanotactic signals, which will be elaborated upon further in the following sections. Protrusions describe membrane extensions in the direction of migration, and two main protrusive structures have been described: filopodia (long, unbranched, parallel actin bundles) and lamellipodia (branched networks of thin, short actin filaments) (Blanchoin et al. 2014). In ameboid migration, actomyosin-based contractility creates pressure and the flow of the cytoplasm towards the uropod. This flow forms spherical membrane expansions, often called “**blebs**” which facilitate the forward movement (Huse 2017). To move, the forces need to be transmitted from the cell membrane to the substratum. In adhesion-dependent migration, such as mesenchymal migration, this process is predominantly mediated by adhesion molecules (integrins) (Ridley et al. 2003). However, immune cells can migrate independent of the integrins (Lämmermann et al. 2008), and instead via unspecific friction forces with the environment (Hawkins et al. 2009). Following the development of protrusions, the cell body translocates, a process that is coordinated by and dependent on myosin II, which together with microtubules, controls the translocation of the nucleus. Finally, for the retraction of the uropod, several mechanisms converge (Capuana et al. 2020; Mayor and Etienne-Manneville 2016); e.g., interplay between microtubule depolymerization, and actomyosin-mediated retraction during DCs migration (Kopf et al. 2020).

All of these migration steps are supported by the three main components of the cytoskeleton: actin filaments, microtubules, and intermediate filaments. In immune cells, the actin cytoskeleton provides protrusive and contractile forces in cooperation with myosin IIA. The microtubule network not only provides tracks for organelles and vesicles to be transported within the cell, but also contributes to maintenance of nuclear morphology. Signaling pathways coordinate the dynamic interactions between the cytoskeletal elements (Devreotes and Horwitz 2015). These elements include, for example, the Rho-family of GTPases (involved in indirect regulation of actin dynamics), actin regulators such as the formins (involved in polymerization of actin), the Arp2/3 complex (involved in nucleation and branching of actin), and members of the WASP/WAVE family (Arp2/3 activators). As the cytoskeletal components can dynamically adapt to the environment, this allows the cells to “squeeze through” small spaces, where the size of the relatively stiff nucleus becomes a decisive limiting factor. Squeezing of the nucleus might induce DNA damage (Denais et al. 2016; Lammerding and Wolf 2016; Raab et al. 2016) if the DNA repair mechanisms are insufficient or defective, therefore limiting cell survival and triggering apoptosis (Denais et al. 2016; Raab et al. 2016). Interestingly, immune cells have multilobed nuclei, which effectively reduces the absolute size of the stiffest object that needs to be squeezed through any constrictions. This property certainly reduces the risk of nuclear rupture and DNA damage during this squeezing of the cell contents (Yamada and Sixt 2019).

The intermediate filaments are responsible for the maintenance of the overall cell shape, and also for the integrity of the nucleus (Danielsson et al. 2018; Hohmann and Dehghani 2019; Huse 2017). The intermediate filament vimentin has a fundamental role in maintenance of nuclear integrity during cell migration (Patteson et al. 2019b), as well as in regulation of cell speed and cell persistence (see Box 1 for definitions) during migration (Patteson et al. 2019). Indeed, epithelial cells treated to switch from keratin to vimentin expression undergo a transition from slow mesenchymal migration to fast amoeboid migration (Lavenus et al. 2020), which supports of the role of vimentin in amoeboid migration. Thus, while vimentin is broadly described as a regulator of mesenchymal migration, recent evidence supports its role equally in immune cell migration.

Box 1 Parameters and properties of cell migration

**Table:** 

**Cell speed** The mean cell speed is defined as the total distance of the cell migration divided by the total acquisition time. The instantaneous speed of migrating cells is calculated for two successive images.	
**Cell persistence** Cell persistence defines the “straightness” of the cell movement, which has different definitions depending on the device geometry used. In one dimension, there is a unique direction, so the persistence length corresponds to the mean length a cell travels before it stops or turns back, which is usually normalized to the channel length. In two dimensions and three dimensions, the cell persistence can be defined as the diameter of the smallest disk containing the whole cell trajectory divided by the total distance of the trajectory. Another common definition is the angular persistence, which also considers the turning angles all along the migration path. In all cases, the persistence scale lies between 0 (non-persistent) and ⌈±1⌉ (highly persistent).	
**Mean first passage time** The mean first passage time is defined as the average time a searching cell takes to find a target, such as another cell (e.g., for procreation, immune synapse formation), a pathogen, or nutrients. This parameter depends on the number and motility of searchers and targets.	

### Challenges and scenarios immune cells encounter in vivo

While migrating through the body, immune cells face various scenarios, which range from one-dimensional (1D) to three-dimensional (3D) environments, and these cells often need to adapt and switch from one environment to another (Fig. 1b). 3D conditions are their most common environment in vivo, such as in peripheral tissues and organs, including the lymph nodes. Migration through 3D environments requires the cells to squeeze through complex extracellular structures with specific cellular adaptation to the mechanical features of the ECM (Yamada and Sixt 2019). Two-dimensional (2D) migration is the best-studied and best-understood form of cell migration in vitro (Ridley et al. 2003). In vivo, 2D immune cell migration can be seen during extravasation when cells roll on, attach to, and crawl along the walls of blood vessels, before they penetrate into the tissue (Filippi 2016; Nourshargh and Alon 2014). The first barrier immune cells encounter is the vessel wall composed of cells (endothelial and pericytes) and a basement membrane. During inflammation, immune cells squeeze in between endothelial cells or through them before transmigrating through the basement membrane. The penetration of cells into the tissues, called diapedesis, might be modified by a reorganization of the basement membrane that can lead to diseases (Friedl and Weigelin 2008; Korpos et al. 2013; Leclech et al. 2020). 1D scenarios are less common physiologically but still present in vivo. The capillaries of the lymphatic or vascular systems have a mean diameter of ~ 5 μm (Henderson et al. 2020). In those capillaries, whether leukocytes actively migrate and how is still not fully understood. Nevertheless, evidence from in vitro microchannel experiments shows that without external sheer force, murine CD8^+^ T cells do crawl in the microchannels with a width of 4 μm or 8 μm (Jacobelli et al. 2010), suggesting that immune cells could migrate actively in these capillaries in vivo. In addition, hydrodynamic forces can further promote leukocyte movement in blood capillaries (Kameritsch and Renkawitz 2020). However, cells proceed along a line or a linear structure that can be considered as 1D migration (Jackson 2019; Nortley et al. 2019). Another example of 1D migration in vivo is during cell movement along ECM fibers, which depends on the local density and alignment of the collagen around the tissue or tumor boundaries (Yamada and Sixt 2019). A recent in vitro study shows that primary human CD8^+^ T cells preferably migrate through the channels formed in collagen matrix (Sadjadi et al. 2020), suggesting another possible 1D scenario for immune cell migration in vivo. Thus, 3D, 2D, and 1D environments are all physiologically relevant conditions, whereby each requires the use of a different migratory mechanism by the immune cells.

### The complexity of the environment shapes the migration of immune cells

As indicated, the environments that immune cells encounter in vivo are diverse, and can have different physical and chemical properties, such as the composition of the ECM, the stiffness and geometry of the tissue, and the presence of chemokines. All of these features collectively define the behavior of immune cells during their migration.

The ECM is defined as all of the noncellular components of tissues and organs. It consists mostly of proteoglycans and fibrous proteins, such as collagen (Lämmermann and Germain 2014). Those noncellular components can vary in composition, and therefore expose the embedded cells to varying surrounding properties, which in turn influences cell migration (Lange and Fabry 2013). Along with stiffness, porosity (the size of pores/channels in the ECM) and geometry are also key physical features of the ECM, and these can also influence immune cell migration. Spatially varying stiffness can be established, e.g., by different concentrations of structural proteins like collagen, and this can result in cell migration up a stiffness gradient, which is referred to as durotaxis. In a physiological context, stiffness gradients have been observed in a number of diseases, such as with lung fibrosis, breast cancer, and atherosclerosis (Hartman et al. 2017). These stiffness gradients have been shown to be a consequence of changes on ECM composition. Such increase in tissue stiffness from the tumor core to the periphery in cancers is believed to favor metastasis and tumor spreading (Hartman et al. 2017).

Another relevant feature that influences cell migration is the geometry of the ECM. The ECM often has a filamentous structure with enough space between fibers for cells to pass through, the size or width of which is largely dependent on fiber density. However, as migrating cells move along, the space occupied by the cells also moves, and the surrounding tissue deforms. The structural properties of the ECM are known to impact upon cell migration, such as, fiber density and organization (i.e., ECM porosity), and ECM protein composition. The path of least resistance with appropriate sizes of pores can thus provide a route for rapid cell passage during in vivo migration. This is especially relevant for immune cell migration, where there is no enzymatic modification of the surrounding ECM (Yamada and Sixt 2019). In addition to the ECM, cell networks can influence immune cell migration, such as the fibroblastic reticular cell network, which can form a structural backbone that actively guides T cell movements inside the lymph nodes.

Chemoattraction describes directed migration patterns towards higher concentrations of chemokines. Chemokines are small proteins that are released by immune, epithelial, and endothelial cells in response to various stimuli, such as tissue injury or infection. These chemokines attract immune cells along the concentration gradient. Chemoattractant-driven migration is termed chemotaxis (or haptotaxis, if the gradient is bound to a substrate), and this has a key role in the regulation of immune cell behavior. For example, expression of the CCR7 chemokine receptor is required for activated DCs to migrate through the lymphatic system (Lämmermann and Germain 2014). The CCL19 and CCL21 chemokines are both ligands for CCR7, but CCL21 is considered to be the critical chemokine for the migration of activated DCs (Worbs et al. 2017). T cell migration is also guided by chemoattractants, such as CCL19, CCL21, and CXCL12, which are required for optimal naïve T cell motility in vivo (Lämmermann and Germain 2014). Chemokines in the lymph node increase basal T cell motility, although they do not appear to contribute to the search strategies undertaken by T cells at the initiation of a response. Although CCR7 is required for T cells to maintain their average speed, it does not control the other features of the random walk, including the directionality (Cotta-de-Almeida et al. 2017).

Fibroblasts are the major cellular component in the ECM, and lymphocytes are in contact with fibroblasts most of the time while they move through the lymph nodes. Therefore, the influence of fibroblasts on T cell migration is also of particular interest for studies of immune cell migration. Nevertheless, the signals that control those interactions remain poorly characterized. One mechanism whereby fibroblasts can guide T cell migration directly is through the creation of channels: e.g., by producing collagen and modifying the ECM, or by releasing cytokines and chemokines that guide T cell movements directly (Bajénoff et al. 2008).

## Migration of immune cells in diverse microfabricated geometries

Understanding the relative roles of free migration versus mechanically or chemically guided cell movements is thus essential for the development of a better picture of how these events are regulated in vivo (Castellino et al. 2006). We summarize now the methods to investigate the effects of such environmental cues on immune cell migration.

**Fig. 2:**
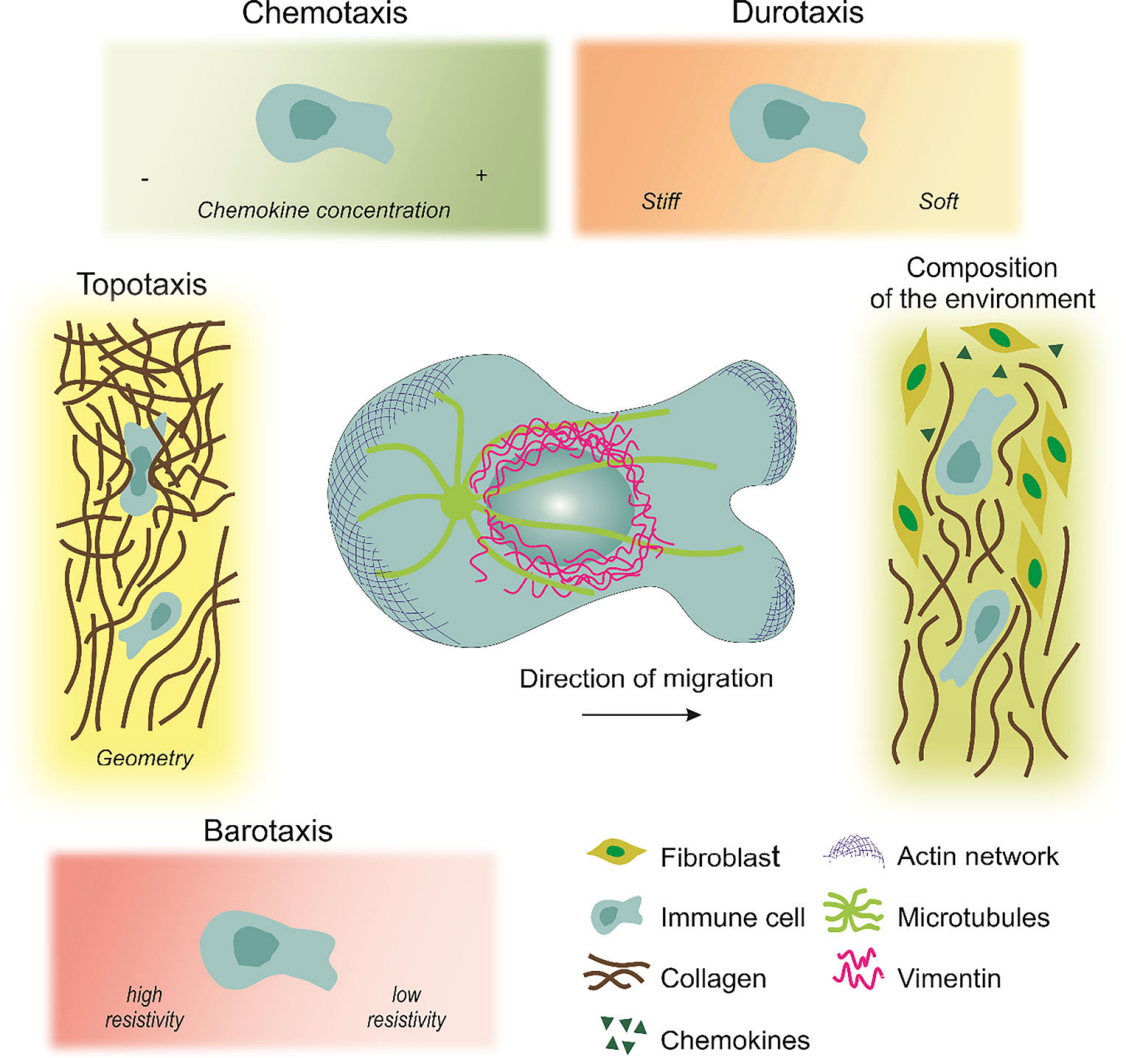
Migratory challenges and guidance cues encountered by immune cells during circulation. The extracellular regulation of cell migration includes: chemokines and stiffness gradients; the extracellular matrix (ECM) mechanics (including loose or highly cross-linked zones) and its topolography (pores, or obstacles); the molecular composition of the matrix surrounding the cells (collagen, fibroblasts, chemokines) as well as pressure gradients. Center: Schematic representation of a polarized immune cell migrating directionally in an amoeboid migration mode. Amoeboid migration is characterized by a round cell morphology, low adhesive contacts and cell body deformation driven by actin protrusions. The microtubule organizing center is generally located at the back of the nucleus. The integrity of the nucleus is protected by a nuclear cage formed by intermediate filament proteins, such as vimentin

Visualization of immune cell migration in vivo is feasible using, e.g., intravital microscopy; however, the interpretation of the results obtained remains difficult. Comparing direct in vivo observations with well-defined in vitro environments (i.e., in terms of geometry, mechanics, chemical and physical cues, see Fig. 2) is essential to go further in our understanding of immune cell migration and immune responses. In this section, we describe how immune cell migration is studied in terms of in vivo to in vitro experiments. We illustrate how and why microfabrication can mimic physiological environments, at least partially, with a focus on different techniques and their implementation, as well as their applications. We analyze the pros and cons of each of these systems, and describe the specific questions they address in terms of immune cell migration (as summarized in Table 1). For general reviews about cell migration, please refer to (Ghibaudo et al. 2011; Lautenschläger and Piel 2013).

**Table 1:**
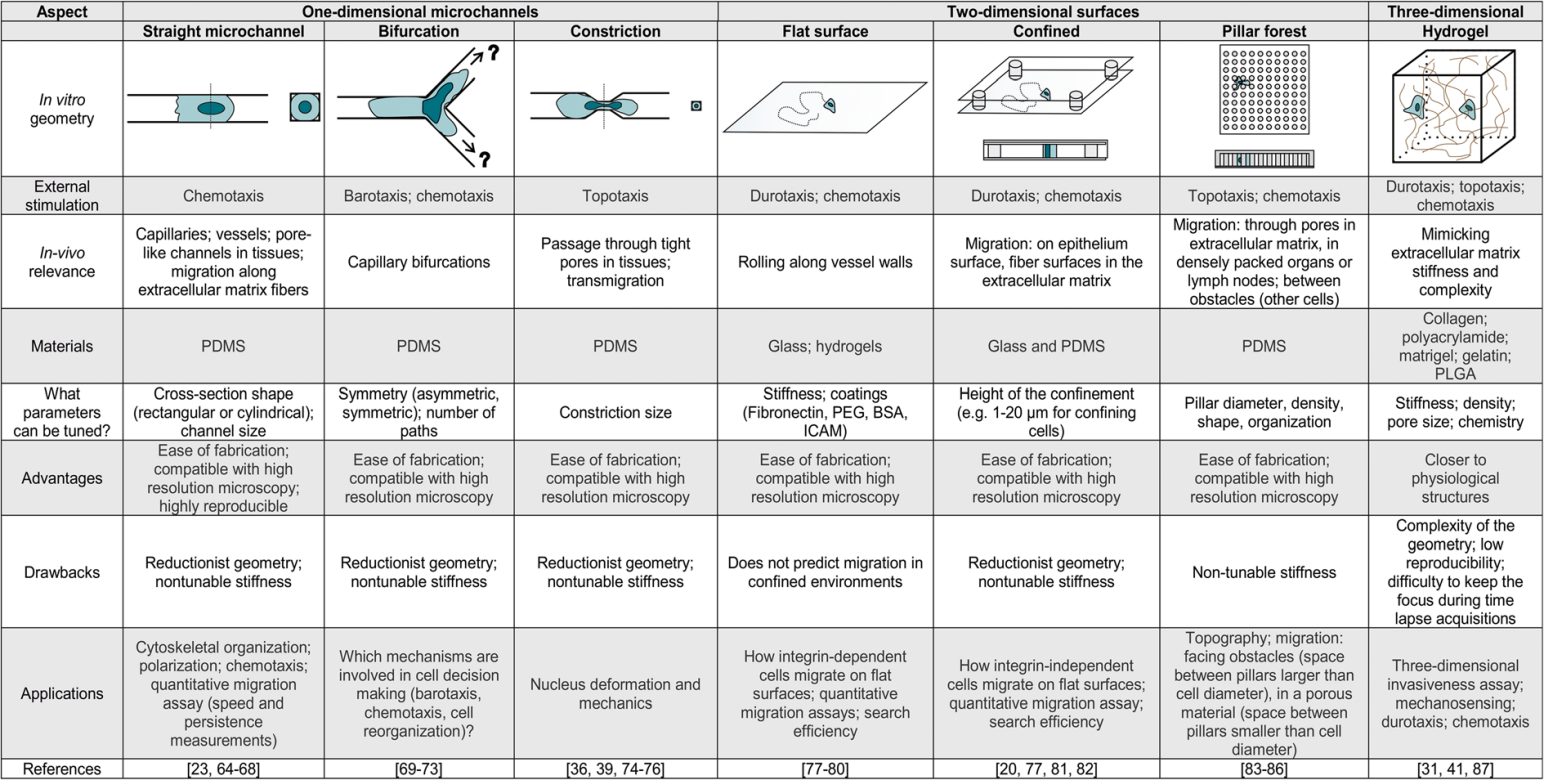
Characteristics, advantages, and drawbacks of various geometries used for in vitro studies

*PDMS*, polydimethylsiloxane; *PLGA*, poly(d,l-lactic-co-glycolic acid); *PEG*, polyethylene glycol; *BSA*, bovine serum albumin; *ICAM*, intercellular adhesion molecule

### Characterization of immune cell migration using in vivo models to build in vitro systems

Intravital microscopy consists of imaging cells of a living animal through a transparent tissue or a transparent window placed in the body by surgery (Murooka and Mempel 2012). This can allow direct observations of immune cell migration in their physiological context, and in various tissues (Weigert et al. 2010). Depending on the experiment and the invasiveness of the surgery, the animal is sacrificed at the end of the experiment. This technique requires specific labeling of the cells, which is usually performed using transgenic animals. However, some parts of the body are not trivial to access in vivo and require ex vivo experiments that externalize a tissue or organ to study it. In vivo*/*ex vivo migration experiments are often performed on mice (Abdul Hamid et al. 2020; Raab et al. 2016) or zebrafish (Barros-Becker et al. 2017; Cougoule et al. 2012; Rosowski 2020), because they are small enough to be positioned under a microscope (e.g., confocal, multiphoton). The observation of the native environment of the cells has inspired the conception and design of in vitro experiments that are closer to the true physiological conditions, and where the effects of single mechanisms can be studied without the influence of other parameters. The advantages of in vitro experiments are that they can be well controlled, and limit the number of animals used for research.

Over the last two decades, the “microfabrication” technique has been used widely to provide reliable, versatile, and reproducible systems with well-defined geometries (Whitesides et al. 2001). To mimic the extracellular environment encountered during in vivo migration, a bottom-up approach has usually been used, where the levels of complexity can be tuned. The simplicity of each device enables exploration of the fundamental mechanisms related to single or collective cell migration that would not have been understood in the complexity of in vivo environments (Garcia-Arcos et al. 2019).

**Fig. 3:**
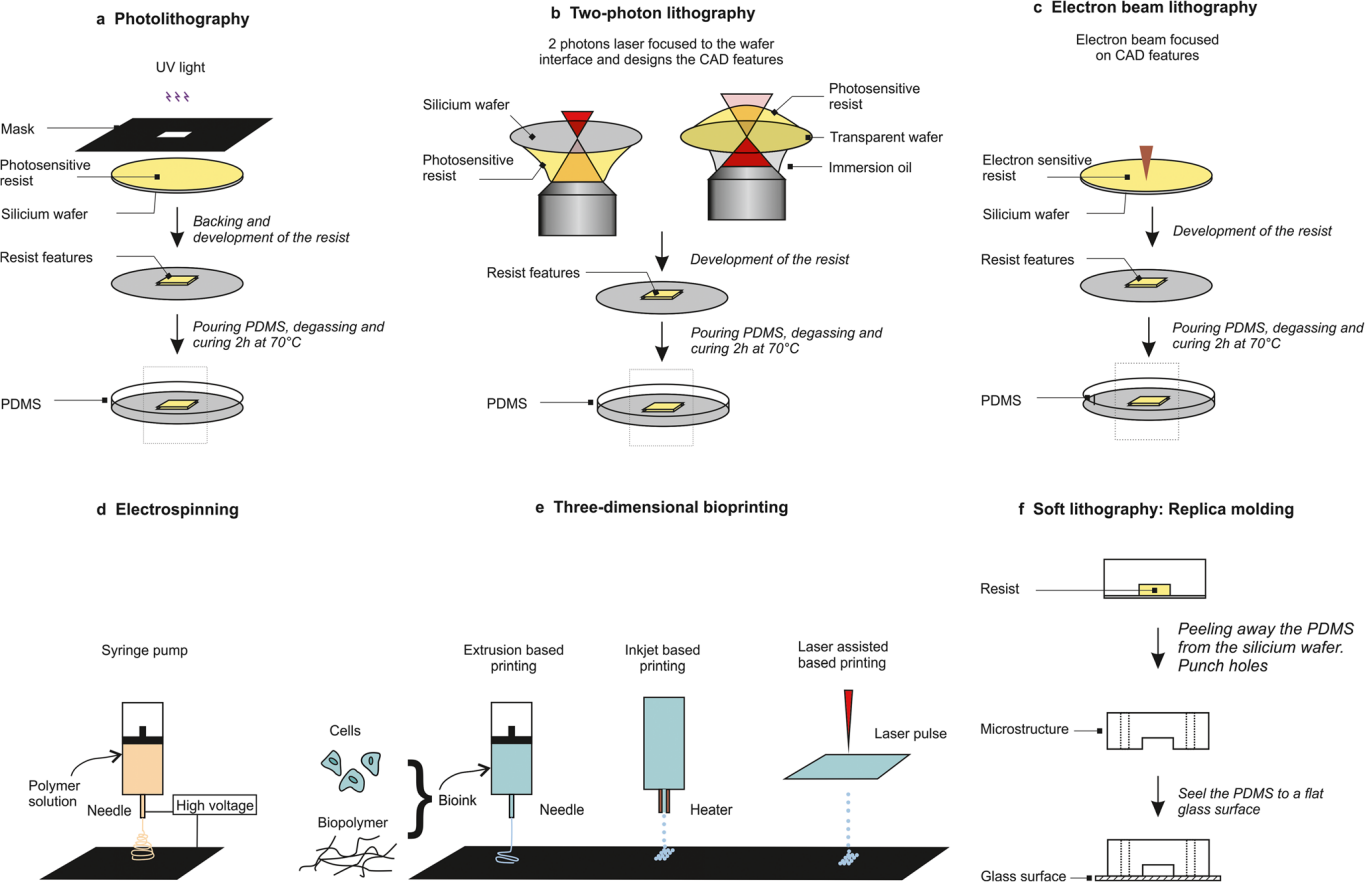
Techniques used to study in vitro cell migration. (a) Photolithography: the photosensitive resist is exposed to UV light through a mask where the features are designed. (b) Two-photon lithography: mask-free technique where the features are directly printed in the resist from the resist–wafer interface with a piezo motion in the three space dimensions (X, Y, Z). The resist is up or down depending on the wafer transparency (image inspired by (Bückmann et al. 2012)). (c) Electron beam lithography: mask-free technique where the features are directly printed by reticulation of the resist in contact with the electron beam. (d) Electrospinning: mask free technique where a polymer solution is extruded from a needle around which a high voltage electric field is formed, and deposited on a surface that can be rotating. The 2D membrane fabricated using this technique can then have random to aligned nanofibrous structures depending on the needle translation. (e) Three-dimensional bioprinting: mask-free technique where a bio-ink consisting of cells and biopolymers is directly deposited on a surface by extrusion-, inkjet- or laser-assisted-based printing. (f) Soft lithography: this step follows the production of the wafer with one of the techniques presented in (a, b, and c); polydimethylsiloxane (PDMS) is peeled away from the wafer, punched, cut, cleaned and bound to a glass surface. CAD, computer-aided design

Microfabrication usually follows two steps. The first step consists of producing the silicon wafer that is the mold for the final device. The main techniques here are photolithography and two-photon lithography. The second step consists of producing the final device that is to be used directly for the experiments. Soft lithography and hydrogel-based systems are usually used for this purpose. More recently, 3D printing has allowed the direct fabrication of these devices with no need for the wafer production. Here, we present an overview of the most commonly applied techniques (Fig. 3) and their respective advantages and drawbacks (Table 2).

**Table 2:** Main characteristics of lithography techniques

Characteristic	Photolithography	Electron lithography and two-photons lithography	Three-dimensional bioprinting
Flatness	Homogeneous height given by the spin-coating speed	Structure can be tilted	Lines can appear due to needle shape
Resolution in xy	Down to ^~^1*μ*m	Down to ^~^100nm	Size of nozzle (100 *μ*m)or droplet (20 *μ*m)
Resolution in z	1–100 *μ*m	^~^100nm	<10 *μ*m
Printing	Mask required	Direct printing, mask-less	Direct printing; mask-less
Equipment	UV lamp (e.g.,UV Kub); spin coater, two hot plates	Nanoscribe	Bioprinter
Costs	Low	High	Low
Comments	Two masks needed for a two-layer devices	Different height can be printed at the same time	Used with different kinds of materials; better for large structures (>500 *μ*m); aims to print scaffold for organs
Example Suppliers	Selba, Rose photomask (for mask); Microfactory, Si-Mat (for wafer); Blackhole (from microfabrication kits to devices)	Nanoscribe’ Semiconductor Production Systems; Heildelberg Instruments	All3DP; Biolife4D; Cellink

#### Photolithography

Photolithography emerged as a technique to respond to the needs for accuracy in the electronics field. It was first described for biological purposes at the beginning of the twenty-first century, by the group of Whitesides (Whitesides et al. 2001). The protocol has been described in detail before (Heuzé et al. 2011; Qin et al. 2010). Briefly, photolithography consists of spin coating a photoresist on the top of a silicon wafer. This photoresist is a light-sensitive material that is used to form a patterned coating on the silicon wafer. A commonly used negative photoresist is the epoxy-based structure known as SU8. The resist is soft baked and then exposed to UV light through a mask (Fig. 3a). The mask can be plastic or chromium-coated glass, which depends on the resolution required and the design features, and can be made using any of the specific computer-aided design software, such as CleWin, AutoCad, and LayoutEditor. Chromium-coated glass masks have the highest resolution (down to ~1 μm), but they are relatively costly. Plastic masks are much cheaper (around €100 for A4 format), and have an XY resolution of about 5 μm. Photoresists are either positive resists, when the UV light exposure makes the resist soluble in the developer, or negative resists, when the UV light exposure initiates the resist cross-linking to make it insoluble in the developer. Therefore, the features of the mask need to be the inverse of the final structure designed for a device when a negative photoresist is used, such as with SU8 or ma-N. The reticulation of the resist is then set by post baking. The last step consists of removing the unreticulated resist using the developer.

Photolithography is the most commonly used technique for microfabrication, and it was built on the expertise developed for the microelectronics field. No specific or expensive equipment is required for photolithography, which is based on only a spin-coater, a UV-lamp, and two hot-plates. This equipment is also not demanding of space. Although photolithography is usually carried out in a clean room, it can be used under a chemical hood in a dark room for structures >5 μm. Ready-to-start sets are commercially available and relatively affordable (<€10,000). Moreover, the homogeneous height of the samples is guaranteed due to the spin-coating step. However, there are also several major limitations in this method. The resolution on the X/Y axes is limited by diffraction, which means that investigation of features below 1 μm is not feasible. Furthermore, the height of the final device is not accurately reproducible in the micrometer range, as it is very sensitive to various external conditions, such as resist viscosity, spinning speed, stability of backing temperature, room temperature (optimal, 20–22 °C), and humidity (usually ~45%). In addition, photolithography is a long process that progresses from the computer-aided design conception to the wafer production over at least several days, and more often, several weeks. For a multi-layer structure, a so-called mask aligner is needed, along with as many masks as the number of layers that are required. Other techniques overcome those limitations by using direct printing, which does not require any mask, saves time, and makes multi-layer fabrication easier.

#### Electron-beam lithography and two-photon lithography

Unlike an indirect method like photolithography, there are numerous direct printing techniques that are available, such as electron-beam lithography (Altissimo 2010) and two-photon lithography (Farsari and Chichkov 2009) (characteristics summarized in Table 2). These mask-free lithography techniques have the advantage that they can reach spatial resolution of about 100 nm, and simple 3D structures of different heights can be printed in one step. Both techniques are adapted to 3D nanofabrication and are very versatile (Niesler and Hermatschweiler 2015). For each scan in the Z direction, the resist/wafer interface has to be precisely determined. To avoid floating structures, the first two to three layers are contained in the wafer; however, this step can induce tilt, in terms of the height (e.g., left side 1 μm higher than right side). For thin structures, a tilt of 1 μm in height can be unacceptable, which will depend on the application. As mask fabrication is not needed, the overall process is considerably shorter compared to photolithography. For example, production of a system with micrometer resolution for an area of several square centimeters normally only takes several hours, except for some complex geometries and/or for very high resolution. The time of printing depends on the balance between the size and the precision for the three space dimensions required.

Two-photon lithography has benefited from the development of femtosecond lasers. In contrast to the standard UV-initiated and mask-based photolithography method, the reticulation for two-photon lithography is initiated locally by two-photon absorption on the area of focus, which is reached with an ultrafast laser (Fig. 3b). Then, as for standard photolithography, the uncured resist (i.e., not exposed to the laser) is removed with the developer.

Electron-beam lithography can achieve a resolution even below 10 nm in all three dimensions (Fig. 3c). However, as the electron beam goes through the material, high aspect ratios in 2D structures are not easy to obtain. Instead of using photosensitive resists, electron-beam lithography uses resists that are sensitive to electron beam radiation (Vieu et al. 2000). The most commonly used electro-sensitive resists are made from polymethyl-methacrylate, as this has the highest spatial resolution (<10 μm) compared to other resists, such as EBR-9 (copolymer of trifluoroethyl-a-chloroacrylate and tetrafluoropropyl-a-chloroacrylate; resolution, ~100 μm), and its fabrication is easier than for polybutene-1-sulfone positive resists (Tseng et al. 2003). Electron-beam lithography is highly reproducible for heights in the range of 10 nm and features from 10 to 1 mm, which makes it useful for patterning (Kolodziej and Maynard 2012). These techniques allow the production of devices for confined migration or migration on 2D surfaces studies but do not allow transmigration studies. For this purpose, other techniques permit in vitro membrane production.

#### Electrospun membranes

Transmigration is a crucial challenge for immune cells as they continuously cross the ECM barrier of the basement membrane. In vivo studies revealed that neutrophils and macrophages migrate across the basement membrane but that neutrophils transmigration is more invasive (Voisin et al. 2009). The basement membrane is a thin densely packed membrane mainly composed of laminin and collagen type VI. It is located around muscles, fat, and in between pericyte cells and endothelial cells in blood vessels (Jayadev and Sherwood 2017). The complex structure of the basement membrane makes it difficult to be studied in vivo*,* therefore in vitro models have been developed (Sobreiro-Almeida et al. 2019). In the early twenty-first century, polymer membranes or Matrigel were used in Boyden chambers to study the invasion ability of cells (Kleinman and Jacob 2001). Even though Boyden chambers were a first step to investigate transmigration, their structure was far from the one of basement membranes. Therefore, new methods emerged to mimic more physiological structure of the basement membrane, such as the electrospinning technique.

The electrospinning technique consists of producing fibrous scaffolds (Kumbar et al. 2008). Thin fibers (in the range of some nanometer to some micrometer in diameter) are produced by applying a high-voltage electric field to a polymer solution. The polymer fibers are extruded through a needle with a diameter that depends on the tip size of the needle, the polymer viscosity, the voltage applied, and the distance between the surface and the needle. The fibers are deposited on the charged rotating or flat surface in a random or parallel organization (Fig. 3d). This technique has the advantages to produce homogeneous fiber sizes. Polymers such as polycaprolactone have been used in order to study immune cells migration through a fibrous membrane of various density (Jin et al. 2015). The 2D structures are then used as a scaffold, and can be coated with collagen for more physiological properties of basement membrane. More recently, electrospinning has been used to directly embed cells into the biopolymers fibers (Hong et al. 2019) and provides promising applications in the field of tissue engineering when coupled with hydrogels solutions and 3D printing.

#### Hydrogel fabrication

To study the influence of the mechanical properties of a substrate during cell migration, 2D and 3D structures have been developed using hydrogels. Hydrogels consist of hydrophilic polymer chains that are mixed in an aqueous phase. The formulations range from synthetic (e.g., poly(d,l-lactic-*co*-glycolic acid), polyethylene glycol, poly-methyl-methacrylate) to natural (e.g., collagen, agarose, alginate, gelatin, chitosan, fibrinogen, hyaluronic acid) origins. The gels are crosslinked by UV light, temperature or chemicals. Hydrogels have the advantage that they can be transparent and biocompatible, like polydimethylsiloxane (PDMS), and they have better free diffusion coefficient for small molecules. Hydrogels can be sensitive to temperature or pH, and their stiffness can be globally tuned, even though their local stiffnesses can vary (i.e., hydrogels can have heterogeneous stiffness). Nevertheless, one major technical issue for hydrogel fabrication is the swelling of the gel after the addition of the medium or other aqueous solutions. For immune cell migration, the reproducibility of accurate shapes for confinement (e.g., pore size) can be challenging.

Hydrogels have been combined with microfluidics for more than 10 years now. To mimic the physiological ECM in terms of stiffness, pore size, and elasticity, more optimization is required for hydrogels (for more detail on hydrogels, see reviews (Goy et al. 2019; Zhang et al. 2016)). Nevertheless, the integration of gels into microdevices has created new opportunities for research that would not have been possible with microfluidics or hydrogels alone, such as long-term chemotaxis using agarose or collagen channels (Cheng et al. 2007; Shin et al. 2011), rapid bacteria responses to antibiotics (Choi et al. 2014), and cancer cell migration (Huang et al. 2018).

#### Three-dimensional printing

The previous methods here are mainly for 1D to 2D structure fabrication. However, reconstruction of a 3D environment is essential to provide a scenario that is close to physiological conditions. Three-dimensional printing is a direct method used to fabricate a desired 3D structure, which offers a reliable approach for the reconstruction of complex 3D geometries using a computer-aided design model, with high reproducibility. This process is termed 3D “bioprinting” when the “ink” used is biocompatible. The initial aim of bioprinting was to produce artificial tissues and organs in vitro (Murphy and Atala 2014), or to regenerate organs in situ (Cui et al. 2017). Three-dimensional bioprinting has also been used for microfluidic applications (Ho et al. 2015): either to build microfluidic chips using bioprinting methods, or to combine 3D bioprinting and microfluidics for fabrication of transplanted organs with better resolution and more complex structures (Ma et al. 2018; Miri et al. 2018). Three-dimensional bioprinting is based mainly on an extrusion process of a bio-ink from a nozzle, which is generally composed of biopolymer gels and cells. This can provide direct fast and simple fabrication, even for complex microfluidic chips (e.g., containing multilayers, valves, mixers). However, it is not yet compatible with devices in the micrometer range, because the spatial resolution depends on the diameter of the printer nozzle and/or the size of the droplets, in terms of using extrusion-based lithography or inkjet lithography, respectively (see Fig. 3e). Currently, the spatial resolution attainable is about 100 μm for the nozzle and 20 μm for droplets (Bishop et al. 2017) (Table 2). Another challenge of bioprinting is that the bio-ink needs to meet many requirements, such as printability (He et al. 2016), optimal viscosity, and optimal gelling time (Colosi et al. 2016), characteristics that vary among the different bio-inks.

In summary, bioprinting is a promising technique to improve our knowledge in the fields of tissue engineering, drug screening, and toxicology testing in organs on chips (Ng and Yeong 2019). It opens new perspectives to investigate cell migration in three dimensions in downscaled artificial organs, or to create new devices of diverse stiffnesses; however, it cannot yet replace standard soft lithography.

#### Replica molding for microfabrication

Replica molding is one of the soft lithography techniques, and it is based on duplication of structures from a mold. It is a versatile technique that follows the production of the wafer (using one of the techniques presented above). In brief, it consists of pouring the PDMS elastomer over the silicon wafer, degassing this in a vacuum chamber, and curing it at 70 °C for 2 h. Then, the PDMS is peeled away from the master, cut, punched, and plasma bound to a glass surface (i.e., slide, dish) (Fig. 3f).

Polydimethylsiloxane has many advantages for the study of cell migration. First, it is biocompatible and nontoxic, which allows experiments on living cells over several hours. Secondly, it is porous, which allows gas exchange with the outer atmosphere and the corresponding CO_2_ proportion necessary to maintain pH 7.4. Furthermore, its transparency makes it compatible with optical microscopy. PDMS is cheap, easy to fabricate, and can be adapted to all geometries. The one major limit of PDMS is that its stiffness is not easily tunable.

### Characterization of immune cell migration when facing external cues

As described in the previous sections, migrating cells face different types of environments in vivo*,* in their physiological context. Immune cells migrate through confined 3D complex ECMs of different stiffnesses and fibrillar densities, and they can face densely packed environments, like in lymph nodes, transmigrating through basement membrane, and within tissues. During their movement around the body, cells can also encounter 2D surfaces (e.g., blood vessel walls, lymphatic vessel walls) and can need to migrate in one dimension (i.e., in capillaries, following a fiber, passing through pores and channels in the ECM) (Fig. 1). As in vivo migration is the result of the combination of the mechanical parameters (e.g., stiffness, porosity gradients), physical parameters (e.g., pressure gradients), and chemical stimulation, it is challenging to dissect out and understand fully the role of individual properties from this complexity, especially for mechanical and physical properties. To study particular aspects of 1D, 2D, and 3D cell migration, many microfabricated structures have been produced to address a number of scientific questions, such as: (1) What is the effect of substrate stiffness on cell migration? (2) What is the minimal constriction (pore diameter) a cell can pass through? (3) What influences cell sensitivity to chemoattractants, pressure, and physical gradients? and (4) What are the key factors that remodel the cell cytoskeleton during migration? In this section, we summarize how external stimulation has been studied in diverse geometries and dimensions, and with the introduction of obstacles to cell searching areas, as examples of more complex environments, where different guidance cues can overlap.

#### Durotaxis

Durotaxis was defined for the first time in 2000 by the group of Wang (Lo et al. 2000), as the mechanical guidance of cell migration from a stiff to a soft substrate. They demonstrated that fibroblasts are more elongated and have a greater spreading area and stronger force generation on stiff compared to soft surfaces. Since then, most studies on durotaxis have been performed using mesenchymal cell migration (Plotnikov et al. 2012; Tse and Engler 2011; Vincent et al. 2013), cancer cell migration (DuChez et al. 2019; Kirmse et al. 2011), and collective cell migration (Spatarelu et al. 2019; Trepat and Fredberg 2011). Many of these studies were performed in two dimensions using different techniques, such as homogeneous stiffness gradients, as alternations of soft and stiff bands (Kuboki et al. 2014) or patterns (Ladoux and Mège 2017). Recently, 3D devices with mechanical gradients have been developed to study cell migration and differentiation, and tissue engineering (Orsi et al. 2017). Several models have been defined to predict cell migration, although these are better suited to 2D migration via focal adhesions rather than to 3D migration (Feng et al. 2019; Harland et al. 2011). Moreover, although mesenchymal cell migration has been shown to follow contact guidance and stiffness gradients, it is still unclear how sensitive amoeboid cells are to durotaxis (Nuhn et al. 2018). As immune cells do not adhere, but instead migrate via pushing forces and rapid deformation, the effects of durotaxis on immune cells have not been as intensively studied.

To confine cells in a controlled manner, PDMS spacers produced using soft lithography are placed between the glass bottom of a dish and its “roof” (Le Berre et al. 2014; Liu et al. 2015) (Table 1). The stiffness of PDMS is much higher than the stiffness encountered by immune cells in their physiological environments. Therefore, a soft confiner made using agarose gel was proposed more recently (Prunet et al. 2020). This has the advantage that it has a stiffness close to the physiological context. Also, tuning the stiffness is a key parameter in the investigation of 3D migration in hydrogels, to better mimic the ECM structure (Nemir et al. 2010; Stowers et al. 2015). It has been shown that activated microglia cells are more sensitive to durotaxis than immature ones (Bollmann et al. 2015), which opens the hypothesis that immune cells might respond to ECM rigidity differently depending on their stage of maturation. To investigate durotaxis in immune cell migration, different hydrogel compositions can be used (see previous section for hydrogel fabrication) to tune stiffness and/or elasticity.

#### Topotaxis

Topotaxis is a term that has been used in scientific publications since the 1940s (Fraenkel and Gunn 1940), but it then referred to stimulus guidance in general (Nossal 1980). It is only since 2016 that topotaxis has been used to exclusively refer to topographical gradients (Park et al. 2016). Here, Park et al. used cancer cell lines where they migrated on top of nanoposts positioned within diverse density gradients. This 2D experiment suggested that the topography of the cell environment has a physical role in directing cell migration. In earlier studies, it had already been shown that cells follow nanoscale microfabricated grooves in vitro (Clark et al. 1991; Wójciak-Stothard et al. 1996), but this was not called topotaxis at the time. Later, topotaxis has also referred to pore-size gradients in one dimension (i.e., channels; Table 1) and to 3D structures (e.g., pillar forests; Table 1). In the literature, the movement from both sparse obstacles to dense obstacles and vice versa has been shown to depend on the ratio between the cell diameter and the space between the pillars. Cells usually tend to migrate through areas where the pore size is comparable to their diameter (Park et al. 2018; Wondergem et al. 2020). As soon as cells encounter obstacles (e.g., other cells, matrix fibers; in vitro: pillar forests), they modify their migration patterns, which initiates topotaxis effects (Schakenraad et al. 2020). In vitro, several constricting geometries have been designed to mimic cell migration through small ECM pores, generally either as a reduction in a channel section (Thiam et al. 2016) or the movement between pillars (Davidson et al. 2014).

In in vitro 1D channels, hydrodynamic forces can be avoided in order to only study spontaneous cell migration comparable with 3D migration. Studies with 1D channels highlight the position and deformability of the nucleus as a limiting factor for cell migration. When DCs are faced with constrictions of ≤3 μm, nuclear deformation can induce the rupture of the lamina envelope (Thiam et al. 2016). Compared to DCs, the nuclei of neutrophils are more deformable (Rowat et al. 2013), which allows these cells to squeeze through pores of <1 μm. Also, for a long time, the effects of external physical cues on cell migration were not studied independently, but mainly in combination with chemotaxis. Recently, it was shown for NK cells that their behavior is modified by topographic effects when following either parallel or perpendicular grooves (Xu and Pang 2019).

Two-dimensional topography has been shown to guide epithelial cells during wound healing (Marmaras et al. 2012). In the context of immune cells, the impact of the 2D topography on their migration still remains elusive. Apart from 2D conditions, immune cells also often face confined environments, which can be mimicked by either adding a roof or a pillar forest to the glass slide on which the cells are plated (Liu et al. 2015). Pillar forests have been developed to study porosity effects on cell migration. These consist of PDMS-based micropillars organized in an array. The pillars touch both the bottom surface and the roof of the set-up (Renkawitz et al. 2018). Depending on the density of the pillars, such a set-up can be used to investigate cell migration through a porous matrix, and to determine the effects of pore size and the presence of obstacles on cell migration (Wondergem et al. 2020). Pillars might represent topographic stimuli that help the directed migration of immune cells, or might act as obstacles that modify the random migration of the cells (Gorelashvili et al. 2014).

#### Barotaxis

Barotaxis refers to migration directions according to pressure gradients. Under physiological conditions, cells often have to choose between different paths (e.g., neutrophils circulation in capillaries (Wang et al. 2020)). The mechanisms that lead cells to take a particular path are not yet fully understood, and barotaxis is being explored as one of these. As hydraulic resistance generates small forces, only amoeboid cells are sensitive to barotaxis, while mesenchymal cells generate high adhesion forces and use proteolysis to migrate. For immature DCs, macropinocytosis limits their sensitivity to barotaxis while exploring any space (Chabaud et al. 2015). During macropinocytosis, the cells take up medium at their front end, which is enough to inhibit the pressure forces. After maturation, DCs lose their ability for macropinocytosis, and instead they polarize and follow hydraulic forces toward the lymph nodes (Moreau et al. 2019). Recently, neutrophils migrating in asymmetric channels were shown to choose the path of least resistance (Prentice-Mott et al. 2013).

Several parameters are suggested to have a role in barotaxis, including cell organization and polarization. In particular, evidence shows that a nucleus-first position (Renkawitz et al. 2019) and microtubules (Ambravaneswaran et al. 2010) act as sensors to facilitate fast migration along the path of least resistance. Moreover, the TRPM7 cation channel has also been demonstrated to be a critical mechanosensor in cell decision-making (Zhao et al. 2019).

#### Chemotaxis

From all external guidance, chemotaxis has been the most intensively studied migration mode of immune cells, since around 1960. Chemotaxis is a general principle that defines a gradient of chemical signals (chemokines, growth factors, substrates or pheromones) and it can be observed in vitro and in vivo (Weber and Sixt 2013). These act as chemoattractants to guide cell migration toward the region with a higher concentration. When the chemical molecules are immobilized on the top of the substrate, the process is called haptotaxis.

Different cell types have specific sensitivities to different chemokines, which depend on their functions and their membrane receptors. This is essential for an efficient search, and to be able to attract the right cells at the right time to the right target. The formation of pseudopodia and the polarization of the cells are the two main responses of cells to chemokines (Van Haastert and Devreotes 2004). Immune cells use both haptotaxis and chemotaxis while patrolling the body, in order to collect information (Schwarz et al. 2017).

Microfluidics is a convenient tool to study chemotaxis in 1D (Prentice-Mott et al. 2016), 2D, or 3D structures. In narrow channels, cells touch all of the walls and block the fluid flow, which allows investigations into the specific impact of drugs or chemicals on one particular side of a cell. Asymmetrical chemical stimulation mimics the chemotaxis in tissues. When the cells sense a chemotactic gradient, they polarize in order to follow it.

The chemosensitivity of cells can be modified by other chemical compounds. It has been shown, for example, that neutrophils lose their sensitivity to N-formyl-L-methionyl-L-leucyl-L-phenylalanine when PI3 kinase is inhibited (which is known to inhibit chemosensitivity in cells). However, it has been demonstrated that this is only true in channels if the PI3K inhibitor is perfused at the front side of the cell, and not at the rear side (Irimia et al. 2007). That suggests that the polarity of the cell and the way it is exposed to different molecules can regulate its chemosensitivity, and thus its migration. Recently, 3D chemotaxis has been studied using microfluidic devices that provide liquid areas with different chemokine concentrations around a solid collagen area. Cells embedded in the collagen can then be exposed to stable chemical gradients in three dimensions (Aizel et al. 2017).

In pillar forests, chemotaxis and topotaxis can be studied together, to understand their respective influences on cell guidance. Cells usually migrate toward a chemoattractant and sparse organizations (Wondergem et al. 2020). It has been shown that if chemical and density gradients are opposing (i.e., higher chemical concentration on the same side as denser pillar organization), then they compete. For example, *D. discoideum* will still migrate toward the chemoattractant, but with a probability to transit toward dense pillars much lower than for the use of aligned gradients (Wondergem et al. 2020). Overall, the cell organization and the response to physical and chemical external stimulation are likely to be the main parameters for all cells to explore their environment in an efficient manner, depending on their functions.

#### Complexity of the ECM: search strategies in a multi-factor environment

In vivo, cells migrate in complex structures such as the ECM, where all biomechanical, biophysical, and biochemical cues compete and enable cells to carry out their functions; e.g., for immune cells to find a target in an optimal time. Although correct immune cell migration is a prerequisite for an efficient immune response, different search strategies guided by all of the different cues indicated above are used to enable the cells to be in the right place at the right time. Many factors are involved in search strategies, including velocity, persistence, turning angle, and mean first passage time (see Box 1 for definitions). Microfabrication is a powerful process for the creation of new geometries and shapes to investigate obstructive systems during cell searching (i.e., “search problematics”). Pillar forests represent one example of structures that are well adapted to this purpose. Different questions can be asked depending on the size of the pillars, their geometrical organization, the height of the device, and the interpillar space. Pillar forests combine the advantages of 2D and 3D structures. First, the visualization of cell trajectories over several hours is easier compared to 3D migration. Secondly, the cell environment is dense and porous, as the cells can encounter many obstacles, which allow immune cells to migrate in an integrin-independent manner. Nevertheless, to date, there have been few studies that have described immune cell migration for diverse pillar organizations. The efficiency of cells during search problematics was initially based on in vivo observations, and then characterized via simulations. We present here an overview of the different search strategies used by immune cells that have been investigated with the help of microfabricated tools.

Emerging evidence shows that cells use different search strategies according to their environment and their functions. It has been reported that immune cells use different types of random and intermittent search patterns (Bénichou et al. 2011). For example, T cells show a random walk (Preston et al. 2006) in vitro but follow a (nonBrownian) Lévy walk in vivo (Krummel et al. 2016), neutrophils are more prone to persistent motion (Jones et al. 2015), while DCs migrate in vitro in an intermittent random walk (Chabaud et al. 2015; de Winde et al. 2020; Worbs et al. 2017). A random walk (i.e., Brownian walk) results in an unpredictable path followed by the cells (Cahalan and Parker 2008; Miller et al. 2002). It has been shown in vivo that DCs adopt a slow random walk in the lymph node with extensive shape change. The fast modification of DCs shape combined with the fast and persistent migration of T cells enables a high number of DC/T cell interactions. It has been estimated that one DC encounters at least 500 different T cells in 1 h (Bousso and Robey 2003). The Lévy and intermittent walks are combinations of an alternation of fast persistent runs and slow, erratic pauses. The main difference between a Lévy walk and an intermittent random walk is the increased possibility of the cell finding a target during the fast motion of the Lévy walk (Moreau et al. 2018). Simulations have demonstrated that cell migration is much more complex and cannot be defined by any single one of these definitions, as it is a combination of all of them (Fricke et al. 2016; Wu et al. 2014). Notably, number of pathogens, number of immune cells, migration speeds, persistence, area to be examined, and time required to find a target all have roles in the efficiency of searching for targets. However, open questions still remain; for example: What is the optimal number of searching immune cells to find a defined number of targets (e.g., pathogens, cancer cells) for the most efficient immune response?

## Conclusions and perspectives

Immune cells are patrolling tissues and vessels to defend our body against pathogens. Failing this task might lead to disease or illness. Therefore, immune cells have to fulfill many roles: (1) they have to find the pathogens, (2) treat the information, (3) transport and convey the information to other cells, (4) these cells then react to this information. In order to fully understand immune cell behavior, in particular immune cell migration, we need to have excellent possibilities to observe and to test parameters of immune cell migration. In this review, we provide a comprehensive summary of microfabrication methods available to investigate immune cell migration. As migration is one of the decisive factors for proper execution of immune cell functions, which is significantly shaped by the environment, here we put a special focus on the context of the challenges (1D, 2D and 3D) imposed to cells migrating in vivo and the respective external regulatory factors (e.g., topography, stiffness, pressure, and chemoattractant). To investigate cell migration in a defined and tuneable way, recently emerging microfabrication has been proven to be powerful tools. Here we summarized the relevant techniques used to investigate cell migration (e.g., photolithography, electron-beam/two-photon lithography, hydrogel fabrication, electrospinning, 3D printing, and replica molding for microfabrication). In addition, we elaborated how the methods can be used to mimic particular aspects of those challenges. So far, we described techniques to test single challenge in static conditions. In living organisms, however, the environment is dynamic and constantly changing. It will be the future of in vitro investigation of immune cell migration to add this dynamic component into artificial environments, e.g., being able to alter the stiffness or geometry of the substrate as well as the chemical available cues while carrying out experiments. Also, several parameters can be combined to study the prevalence of one challenge, e.g., are the cells more sensitive to chemotaxis or topotaxis? In order to understand more complex immune cell behavior, e.g., the search efficiency, that is crucial in the future to develop dynamic experiments in devices that are combining several chemical, mechanical and physical challenges. Optogenetic tools are promising candidates to achieve such goals.

## Data Availability

Not applicable.
